# Electronic laboratory-based reporting: opportunities and challenges for surveillance.

**DOI:** 10.3201/eid0707.017717

**Published:** 2001

**Authors:** D B Jernigan

**Affiliations:** Centers for Disease Control and Prevention, Atlanta, Georgia 30333, USA. djernigan@cdc.gov


### Electronic Laboratory-Based Reporting: Opportunities and Challenges for Surveillance

**Daniel B. Jernigan**
Centers for Disease Control and Prevention, Atlanta, Georgia, USA

Public health surveillance has been defined as the ongoing, systematic collection, analysis, interpretation, and feedback of outcome-specific data used for public health practice (1). Laboratory reports are critical to public health surveillance because they initiate investigations of cases of reportable diseases or outbreaks of infections. The current system of laboratory reporting, which often relies on paper reports delivered by mail, is slow and incomplete. Electronic laboratory-based reporting (ELR) is likely to be more timely and complete (2). A number of challenges must be addressed before ELR can be used effectively, but recent activities are encouraging, and new opportunities for ELR implementation have arisen.

### ELR Challenges

The laboratory landscape is changing. Large national and regional laboratories have developed advanced information technology (IT) capabilites and use standardized test codes, making ELR possible. However, many smaller laboratories do not have the technology necessary for ELR. Additionally, many states have reporting regulations that are not structured for electronic reporting, and health department staff often have limited knowledge of electronic data interchange technology. In the past, public health agencies have focused more on epidemiology and statistics, and less on IT.

### ELR Activities

In 1997, the Centers for Disease Control and Prevention (CDC) and other public health partners sponsored a meeting at which standards for pilot implementation of ELR were determined (3) (i.e., the electronic message format uses the national clinical information standard Health Level Seven [HL7](4), tests are coded with the Logical Observation Identifiers Names and Codes [LOINC] (5), and results are coded with the Systemized Nomenclature for Medicine [SNOMED](6) terminology. At a follow-up meeting in 1999 with broader participation from informatics specialists and representatives from the laboratory community, the group affirmed the ELR approach and planned further implementations (7).

ELR pilot activities have provided valuable lessons. In Hawaii, ELR increased the number of reports 2.3 times, reports arrived 4 days earlier, and demographic data were more complete (2). In Washington and Texas, the feasibility of using HL7 with standardized codes was demonstrated. In Minnesota and Oregon, commercial off-the-shelf messaging software has been used to receive standard electronic messages from national laboratories. Other projects are under way at various sites.

Recently, CDC initiated the National Electronic Disease Surveillance System (NEDSS) to improve public health surveillance through enhanced IT infrastructure and capability. Electronic messaging for clinical and laboratory reports is one of eight elements that will be implemented by using the NEDSS information architecture (8).

Address for correspondence: Daniel B. Jernigan, Office of Surveillance, National Center for Infectious Diseases, Centers for Disease Control and Prevention, 1600 Clifton Rd., Mailstop D59, Atlanta, GA 30333, USA; fax: 404-371-5445; e-mail: djernigan@cdc.gov

### ELR Opportunities

A few large national laboratories contribute a substantial proportion of reports to health departments. Focusing on these laboratories and ensuring their ELR capacity early will likely produce important results. Some of these laboratories can report from a single data repository, which may allow more efficient and possibly enhanced surveillance. Another component of the NEDSS architecture, Web-based reporting, may permit smaller laboratories to more easily participate in ELR. CDC is working closely with standards organizations to ensure that public health needs are represented in national data standards.

### Conclusion

Effective use of ELR will require overcoming several challenges: standardizing message format and coding, developing policy, and improving knowledge and skills for implementation. As part of the larger surveillance system integration initiative of NEDSS, ELR will likely be more accessible to public health partners and providers of public health reports. Opportunities exist for developing public-private partnerships with national laboratories. Public health issues are being incorporated in national standards development. NEDSS support to states will identify current ELR capabilities and provide an opportunity for health officials to examine IT issues.

### References

1. Pinner RW, Jernigan DB, Sutliff SM. Electronic laboratory-based reporting for public health. Mil Med 2000;165 Suppl 2:20-4.
2. Effler P, Ching-Lee M, Bogard A, Ieong MC, Nekomoto T, Jernigan D. Statewide system of electronic notifiable disease reporting from clinical laboratories: comparing automated reporting with conventional methods. JAMA 1999;282:1845-50.
3. Centers for Disease Control and Prevention. Electronic reporting of laboratory data for public health. [document online] 1997 [cited 2000 Oct 1]. Available from: URL: http://www.cdc.gov/od/hissb/docs/elr-1997.pdf
4. Health Level Seven. What is HL7? [document online] 2000 [cited 2000 Oct 1]. Available from: URL: http://www.hl7.org/about
5. Regenstrief Institute. LOINC and Relma. [document online] 2000 [cited 2000 Oct 1]. Available from: URL: http://www.regenstrief.org/loinc/loinc_information.html
6. SNOMED International. Systematized Nomenclature of Medicine. [document online] 2000 [cited 2000 Oct 1]. Available from: URL: http://www.snomed.org
7. Centers for Disease Control and Prevention. Electronic reporting of laboratory information for public health. [document online] 1999 [cited 2000 Oct 1]. Available from: URL: http://www.cdc.gov/od/hissb/docs/elr-1999.pdf
8. Centers for Disease Control and Prevention. National Electronic Disease Surveillance System (NEDSS)—Systems Architecture Elements. [document online] 2000 [cited 2000 Oct 1]. Available from: URL: http:// www.cdc.gov/od
